# Attribution of NKG2DL to the inhibition of early stage allogeneic tumors in mice

**DOI:** 10.18632/oncotarget.10693

**Published:** 2016-07-19

**Authors:** Li Hua, Mingli Fang, Boqi Dong, Sheng Guo, Cuiyun Cui, Jiwei Liu, Yun Yao, Yue Xiao, Xin Li, Yunjia Ren, Xiuping Meng, Xu Hao, Peiyan Zhao, Yilan Song, Liying Wang, Yongli Yu

**Affiliations:** ^1^ Department of Immunology, College of Basic Medical Sciences, Norman Bethune Health Science Center, Jilin University, Changchun, Jilin 130021, China; ^2^ Department of Molecular Biology, College of Basic Medical Sciences, Norman Bethune Health Science Center, Jilin University, Changchun, Jilin 130021, China

**Keywords:** NKG2DL, allogeneic tumors, early stage, NK cells, NKG2D^+^ cells

## Abstract

Allogeneic tumors are eventually rejected by adaptive immune responses, however, little is known about how allogeneic tumors are eradicated at the early stage of tumor development. In present study, we found that NKG2DL low expressing cancer cells were developed into palpable allogeneic tumors in mice within a week after the inoculation, while NKG2DL high expressing cancer cells failed to. The NKG2DL high expressing cancer cells could increase NKG2D^+^ NK cells in the allogeneic mice after being inoculated for 3 days. Artificially up-regulating NKG2DL on cancer cells with low level expressed NKG2DL by a CpG ODN resulted in the retardation and rejection of the allogeneic tumors at the early stage. The contribution of up-regulated NKG2DL to the early rejection was further confirmed by the results that the development of allogeneic tumors from cancer cells transfected with NKG2DL genes was significantly inhibited in mice at the early stage. Overall, hopefully, the data may provide insights for combining the allogeneic NK cell adoptive transfer with the approaches of up-regulating NKG2DL to treat cancer patients.

## INTRODUCTION

In allogeneic mice, not all types of tumor cells initiate early stage tumors after inoculation, while the tumors, if initiated, are unavoidably regressed at later stage. The regression is mainly resulted from the cellular immune response to the allogeneic tumor cells. After inoculation, allogeneic tumor cells, much like the cell in transplanted allogeneic organs, are recognized by both of CD8^+^ T cells and CD4^+^ T cells. The CD8^+^ T cells recognize MHC-class I molecules on the allogeneic tumor cells and are activated to proliferate and differentiate into effector cytotoxic T lymphocytes (CTLs) at the later stage of allogeneic tumor formation. The effector CTLs move to the allogeneic tumors and kill the tumor cells [1]. CD4^+^ T cells are activated after recognizing MHC class II molecules on allogeneic cells [2] to launch adaptive immune responses, resulting in directly killing or promoting the generation of allogeneic cell specific CTLs [3, 4]. Recently, depletion of CD4^+^ or CD8^+^ T cells prior to allogeneic tumor inoculation was found to prevent eventual tumor regression in mice, confirming that CD4^+^ or CD8^+^ T cells mediate the allogeneic tumor regression at the later stage [5]. The tumor-reactive T cells were activated by dendritic cells (DCs) that internalized allogeneic tumor antigens with facilitation by naturally occurring tumor-binding IgG antibodies [5]. Conclusively, eradicating allogeneic tumors is mediated by adaptive cellular immune response at the later stage. However, up to now, no research has been reported on why some types of tumor cells could and others could not develop into allogeneic tumors at the early stage, and on what mechanisms are underlying the phenomenon. Noticeably, depletion of natural-killer (NK) cells was revealed to accelerate allogeneic tumor formation in mice at the early stage, although the depletion did not prevent the allogeneic tumor rejection at the later stage [5]. The data imply that NK cells could mediate the early stage elimination of the allogeneic tumor.

NK cells form a first line of defense against tumor cells [6] and the responsiveness is modulated by engagement of activating receptors, such as NKG2D, on the NK cells with the activating ligands, such as NKG2D ligands (NKG2DL) on the tumor cells [7, 8]. Except NK cells, subsets of CD8^+^ T cells, γδ T cells and NKT cells are also expressed NKG2D, and the NKG2D expressing cells are identified as NKG2D^+^ cells [9]. In mice, NK cells reject tumor cells with up-regulated NKG2DL including retinoic acid early inducible gene 1 (RAE-1)-like proteins (RAE-1α, β, γ, δ, ε), members of the H60 protein family (H60a, b, c) and murine UL16-binding protein-like transcript 1 (MULT-1). In human, the counterpart NKG2DL are MHC class I chain-related proteins A and B (MICA and MICB) and UL-16 binding protein families (ULBPs 1-6) and also induce the tumor cell killing by NK cells [10]. The interaction of NKG2D and NKG2DL has a role in immunosurveillance of spontaneous autologous tumors. In syngeneic mice, NKG2DL-transduced tumor cells could be rapidly rejected and failed to develop into detectable tumors [11], and mice deficient in NKG2D exhibited a higher incidence or greater severity of tumors [12]. In human, shedding soluble NKG2DL from the tumor cells might decoy NK cells and be correlated with poor prognosis in patients with melanoma and prostate cancer [13]. Noticeably, the NKG2D-NKG2DL interaction was participated in rejecting allogeneic graft cells, evidenced by that RAE-1 expressing allogeneic bone marrow cells [14] and neural precursor cells [15], after being transplanted in mice, were rejected by NK cells. The interaction was also involved in killing allogeneic malignant cells, exemplified by that MICA/B over-expressing allogeneic glioma cells [16] and breast cancer stem cells [17] were killed by NK cells *in vitro*. However, it remains unclear whether the NKG2DL expression is involved in rejection of allogeneic tumor cells *in vivo*, especially at the early stage after the tumor inoculation.

In the present study, we found that NKG2DL expression levels determined the rejection of allogeneic tumors from breast cancer cells, melanoma cells and glioma cells at the early stage after being inoculated in mice, and NKG2DL up-regulation led to the early rejection by activating NKG2D^+^ NK cells and NKG2D^+^ CD8^+^ T cells. The study reveals why and how the allogeneic tumor cells are rejected at the early stage, and may provide insights for reinforcing the efficacy of allogeneic NK cell treatment of cancers by up-regulating NKG2DL expression.

## RESULTS

### The correlation of the expression level of NKG2DL on tumor cells and their early tumor formation in allogeneic mice

**Figure 1:**
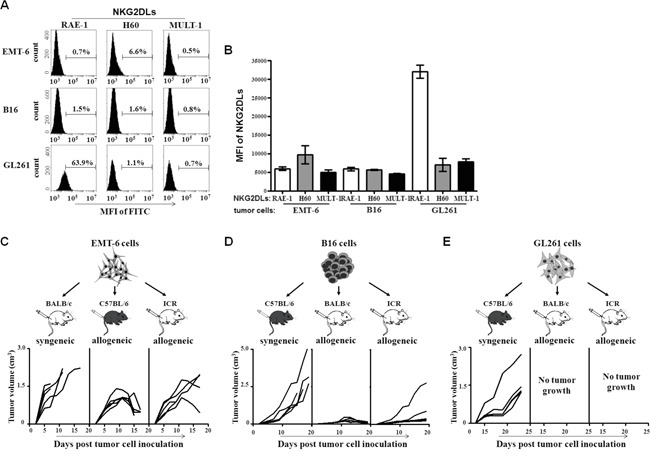
NKG2DL expression and allogeneic tumor formation of EMT-6, B16 and GL261 cells The EMT-6, B16 and GL261 cells were stained with anti-RAE-1, H60 or MULT-1 mAb, respectively, followed by FITC-labeled secondary mAb staining for analyzing their NKG2DL expression. The cells were inoculated s.c. into BALB/c, C57BL/6 and ICR mice, respectively, for observing the tumor formation in syngeneic or allogeneic mice. **A.** The expression levels of RAE-1, H60 and MULT-1 on the tumor cells. One of three experiments was shown. **B.** The graphical presentation of the pooled values from three independent experiments. Each bar represents as means±SEM. The volume of tumors formed by EMT-6 cells **C.**, B16 cells **D.**, and GL261 cells **E.** Each line represents tumor volume in one mouse.

In order to investigate whether the low or high expression of NKG2DL on the tumor cells could determine the tumor formation and rejection in allogeneic mice, we selected three strains of mouse-derived tumor cells, EMT-6 cells (BALB/c mouse origin), B16 cells (C57BL/6 mouse origin) and GL261 cells (C57BL/6 mouse origin) and tested expression levels of the NKG2DL including RAE-1, H60 and MULT-1 on the cells by flow cytometry. As shown in Figure 1A and 1B, the EMT-6 cells were composed of 0.7% RAE-1^+^ cells, 0.5% MULT-1^+^ cells, and 6.6% H60^+^ cells. Similarly, the B16 cells were comprised of low percentage of RAE-1^+^, H60^+^ and MULT-1^+^ cells with an approximate average below 2%. Nevertheless, among the GL261 cells, there were 56.9% RAE-1^+^ cells, 10-fold and 30-fold higher than that in EMT-6 cells and B16 cells, respectively (*P*<0.0001). Moreover, the percentage of H60^+^ cells (0.8%) and MULT-1^+^ cells (0.7%) in the GL261 cells was similar to those in EMT-6 cells and B16 cells. Then, we subcutaneously inoculated NKG2DL low expressing EMT-6 cells, NKG2DL low expressing B16 cells, and NKG2DL high expressing GL261 cells into their syngeneic and allogeneic mice, respectively. On day 5 after the inoculation, the EMT-6 cells began to form palpable tumors in all their syngeneic BALB/c mice (n=5), which circumvented 4 mice by day 13. The remaining one developed a tumor exceeded 2000mm^3^ in size by day 17 and died on day 23. Surprisingly, on day 5 after the inoculation, EMT-6 cells initiated palpable tumors in their allogeneic C57BL/6 mice. The tumors continued to grow and reached to an average volume of 1000mm^3^ on day 9, then retarded and regressed gradually without any treatment, and became impalpable in 4 out of the 5 mice on day 25. Similarly, in their allogeneic ICR mice, the EMT-6 cells also formed palpable tumors on day 5. The allogeneic tumors began to regress on day 11, and could not be palpated in 2 out of the 5 mice on day 25 (Figure 1C). Nevertheless, the allogeneic tumors continually grew and eventually killed the remaining 3 mice on day 30, 41, 53, respectively (data was not shown). Consistently, the NKG2DL low expressing B16 cells also formed allogeneic tumors in both of their allogeneic BALB/c mice and allogeneic ICR mice. In the BALB/c mice, the B16 cells formed palpable allogeneic tumors on day 10. The tumors continued to grow, reached to an average volume of 300mm^3^ on day 19 and then regressed spontaneously. On day 21, 4 out of the 5 BALB/c mice were free of the tumors. In the ICR mice, the NKG2DL low expressing B16 cells formed palpable allogeneic tumors on day 9. The tumors continued to grow, and reached to an average size of 1000mm^3^ on day 19, and then regressed and became impalpable in 4 out of the 5 mice on day 23. As expected, the B16 cells formed continuously growing tumors in their syngeneic C57BL/6 mice (Figure 1D). However, unlike NKG2DL low expressing EMT-6 cells and B16 cells, the NKG2DL high expressing GL261 cells could only formed subcutaneous tumors in their syngeneic C57BL/6 mice, not in their allogeneic BALB/c mice and allogeneic ICR mice (Figure 1E). Upon the subcutaneous model, we further intracranially inoculated the GL261 cells into their syngeneic and allogeneic mice, respectively. Since the developed tumors if excited (Supplementary Figure S2C) could not be grossly observed, we used the body weights and the survival time as parameters to reflect the rejection of the GL261 cells. The results showed that the intracranial inoculation significantly reduced the body weights of the C57BL/6 mice from day 4 after the inoculation, whereas, did not affect the body weight gain in the BALB/c mice and the ICR mice (Supplementary Figure S2A). From day 22 after the inoculation, the C57BL/6 mice began to die of the inoculated glioma cells. By day 30, all of the six C57BL/6 mice died. Comparatively, the BALB/c mice survived significantly longer, began to die from day 28, and died out by day 42 after the inoculation. Notably, 2/6 of the ICR mice died of the inoculation on day 28 and day 32, respectively, and the remaining 4 mice still survived until day 50, showing no symptoms of diseases (Supplementary Figure S2B). Obviously, the data revealed a discrepancy between the NKG2DL highly expressed tumor cells, GL261 cells and the NKG2DL lowly expressed tumor cells, EMT-6 cells or the B16 cells, in their tumor genetic capacity in allogeneic mice, especially at the early stage after the tumor cell inoculation, implying that high expression of NKG2DL on tumor cells may determine the early tumor rejection in the allogeneic mice and NKG2D^+^ cells may be involved in the process rejection.

### The increase of the percentage of NKG2D^+^ cells in allogeneic mice inoculated with NKG2DL high expressing tumor cells

**Figure 2:**
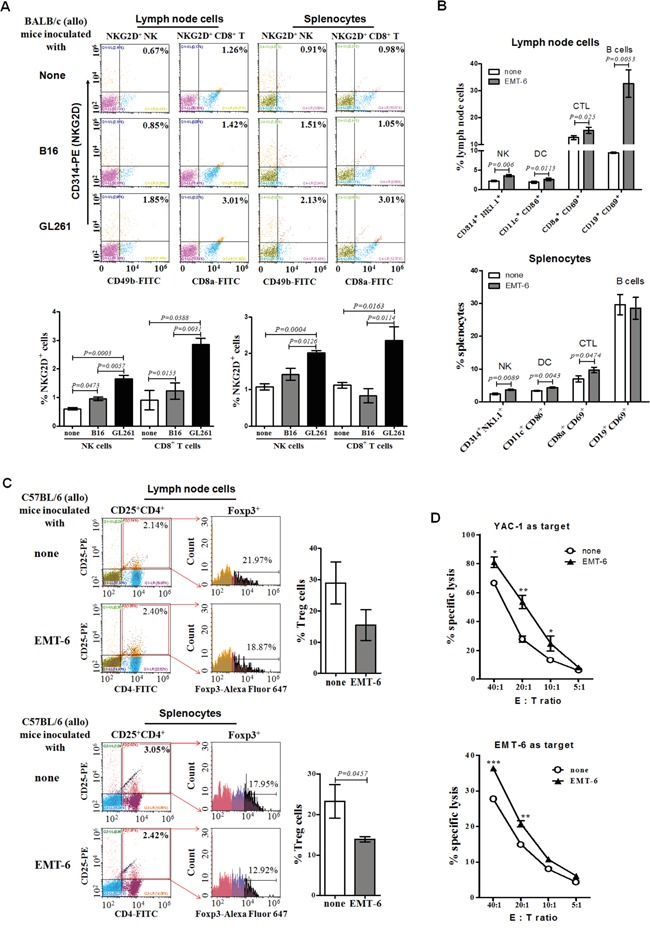
Percentages and cytotoxicity of lymph node cells and splenocytes from allogeneic tumor-bearing mice The lymph node cells and splenocytes from the BALB/c mice inoculated with the B16 cells or GL261 cells for 3 days (early stage), or from the C57BL/6 mice inoculated with EMT-6 cells for 50 days (late stage), were detected for the percentage of NKG2D^+^ NK cells, NKG2D^+^ CD8^+^ T cells, DC, CTL, B cells or Treg, respectively, by flow cytometry. All data were expressed as means ± SEM. **A.** Percentage of NKG2D^+^ NK and NKG2D^+^ CD8^+^ T cells at early stage. **B.** Percentage of NKG2D^+^ NK cells, DC, CTL, B cells at later stage. **C.** Percentage of Treg cells at later stage. The lymph node cells from the C57BL/6 mice inoculated with EMT-6 cells for 50 days were incubated with the EMT-6 cells or YAC-1 cells for 24 hours, respectively, assaying their cytotoxicty **D.** to the EMT-6 cells or YAC-1 cells by fluorescence spectrophotometer by staining with PI. ***<0.0001, **<0.01, *<0.05.

To study whether NKG2D^+^ cells participated in the rejection of allogeneic tumors in mice at the early stage, we analyzed the percentage of NKG2D^+^ NK cells and NKG2D^+^ CD8^+^ T cells in the spleens and lymph nodes of the allogeneic BALB/c mice inoculated with C57BL/6 derived GL261 cells and B16 cells for 3 days, and found that the NKG2D^+^ NK cells and the NKG2D^+^ CD8^+^ T cells in the lymphoid organs of the allogeneic mice inoculated with GL261 cells were a 1.5-fold and 3-fold increase, respectively, compared to that from the mice inoculated with B16 cells (Figure 2A). Noticeably, the inoculation with the EMT-6 cells increased the percentage of the NKG2D^+^ NK cells, but not NKG2D^+^ CD8^+^ T cells, in the spleens and lymph nodes of C57BL/6 mice with complete regression of the tumors for 1.5-fold on day 50 (Figure 2B), and the lymph node cells vigorously killed YAC-1 cells, typical murine NK target cells (Figure 2D, upper panel). On the same day, the percentage of the CTL (CD8^+^ CD69^+^ cells), DC (CD11c^+^ CD86^+^ cells) and B cells (CD19^+^ CD69^+^ cells) was 1.3-fold, 1.5-fold and 3.5-fold higher in the lymph nodes of the allogeneic C57BL/6 mice with complete regression of the tumors than that of naive mice (Figure 2B, upper panel), and the percentage of the regulatory T cells (Treg, CD4^+^ CD25^+^ Foxp3^+^ cells) tended to decrease in the mice (Figure 2C, upper panel). The lymph node cells from the C57BL/6 mice specifically killed the EMT-6 cells *in vitro* (Figure 2D, lower panel). Similarly, in the spleens of the mice, the percentage of the CTL (*P*=0.0474) and DC (*P*=0.0043) were increased (Figure 2B, lower panel), and Treg (*P*=0.0457) were decreased (Figure 2C, lower panel).

### The correlation of up-regulated NKG2DL and tumor inhibition in syngeneic mice

**Figure 3:**
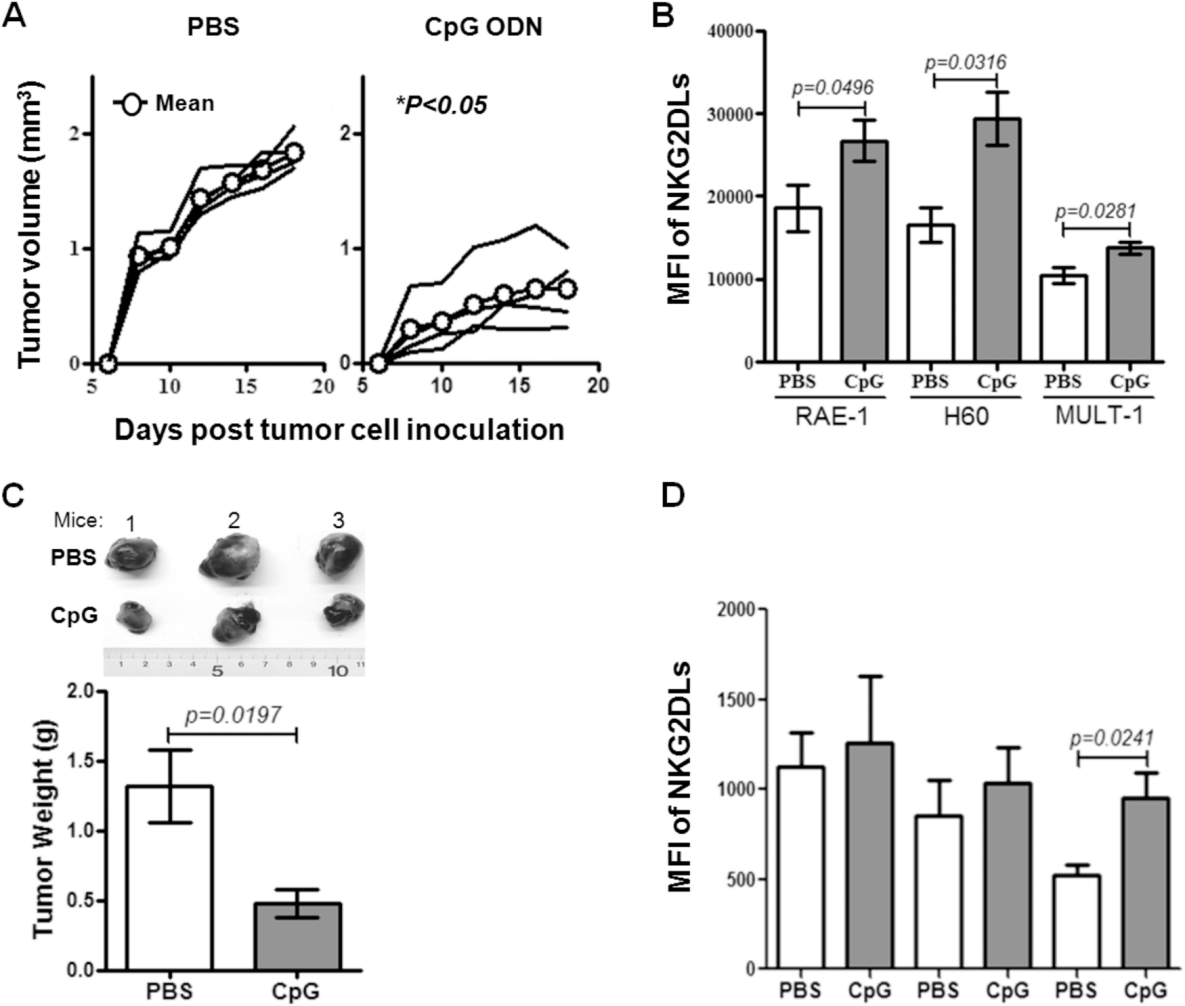
The development and NKG2DL levels of syngeneic tomor The BALB/c or C57BL/6 mice were inoculated with EMT-6 or B16 cells on day 0 and then treated with the CpG ODN after tumor palpated. The tumor development was observed and the NKG2DL expression on the tumor cells was analyzed by flow cytometry using anti-RAE-1, H60 and MULT-1 mAb. **A.** The growth curve of EMT-6 cell-derived tumors in BALB/c mice. Each line represents tumor volume in one mouse. **B.** NKG2DL levels on the EMT-6 cell-derived tumor cells. **C.** The photos and weights of B16 cell-derived tumors. **D.** NKG2DL expression levels on the B16 cell-derived tumor cells. Data from three mice are shown and represented as means±SEM.

To further find the correlation between the expression of NKG2DL on tumor cells and the tumor rejection, we observed the tumor development in syngeneic mice injected with a CpG ODN, a synthetic oligodeoxynucleotide containing CpG motifs which was found to induce regression of the EMT-6 cells derived established breast cancer tumors and recruitment of the NK cells to the tumor site in syngeneic BALB/c mice in our previous work [18]. We inoculated the EMT-6 cells into the syngeneic BALB/c mice, and observed the tumor development by measuring the tumor size every other day when the tumors were palpable on day 5. From the day, we treated the mice with the CpG ODN for six times in one-day interval at tumor draining lymph node (TDLN) area. After 48 hours of the last injection, tumors were removed for analyzing the expression of NKG2DL on the tumor cells by flow cytometry. Consistent with our former observation [18], the tumors of PBS-treated mice were an average 2-fold larger, with approximately 1000mm^3^ in an average volume by day 18, than those of the CpG ODN-treated mice (Figure 3A). In parallel, the expression of RAE-1 (*P*=0.0496), H60 (*P*=0.0316) and MULT-1 (*P*=0.0281) on the tumor cells from CpG ODN-treated mice were an average 1.5-fold increase, compared to those in PBS-treated mice (Figure 3B). Together with the previous work, we proposed that the up-regulation of NKG2DL on the NKG2DL low expressing EMT-6 cell-derived tumor cells caused the breast cancer tumor regression in syngeneic mice. To confirm the proposition, we inoculated s.c. another NKG2DL low expressing B16 cells into C57BL/6 mice followed by treating with CpG ODN as described above from day 10 when the tumors were palpable. After 48 hours of last injection, the tumors were removed to weigh and the tumor cells were analyzed for NKG2DL expression. The results showed that the CpG ODN-treatment significantly decreased the tumor size (Figure 3C, upper panel), and the tumor weight by an average 3-fold (*P*=0.0197) (Figure 3C, lower panel). In parallel, compared with those from PBS-treated mice, the expression of MULT-1 (*P*=0.0241) on the tumor cells from CpG ODN-treated mice were 2-fold increase, while no obvious change in the expression of RAE-1 and H60 on the tumor cells (Figure 3D).

### Up-regulation of NKG2DL on the NKG2DL low expressing tumor cells *in vitro*

**Figure 4:**
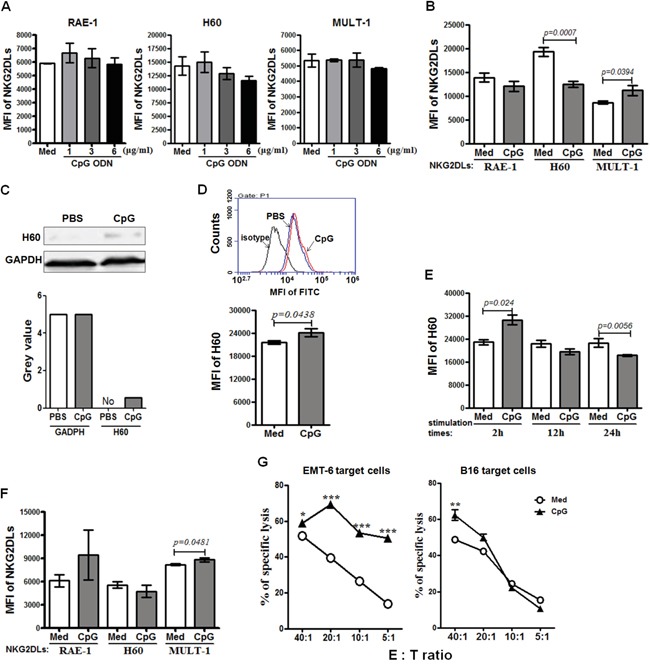
The susceptibility of NKG2DL up-regulated tumor cells to cytotoxicity EMT-6 and B16 cells were cultured with CpG ODN or CpG ODN-conditioned supernatant for 24 hours for analyzing their NKG2DL expression by flow cytometry using anti-RAE-1, H60 or MULT-1 mAb or by western blot using anti-H60 mAb. Data are represented as means±SEM of three independent experiments. NKG2DL expression on the EMT-6 cells stimulated by the CpG ODN **A.** or the CpG ODN-conditioned supernatant **B.** Total H60 expression of the EMT-6 cells stimulated with the CpG ODN-conditioned supernatant, detected by western blot **C.** or flow cytometry **D. E.** The surface expression of H60 on the EMT-6 cells stimulated by the CpG ODN-conditioned supernatant at various time points. **F.** NKG2DL expression on the B16 cells stimulated by the CpG ODN-conditioned supernatant. The EMT-6 or B16 cells were co-cultured with lymph node cells from naive BALB/c or C57BL/6 mice in the presence of CpG ODN at 6μg/ml for 24 hours for assaying their susceptibility to the lymph node cell killing **G.** by fluorescence spectrophotometer after staining with propidium iodide (PI). Percentages of specific lysis are shown for different E:T ratios. ***<0.0001, **<0.01, *<0.05.

Upon the *in vivo* results, we further studied whether the CpG ODN could up-regulate the expression of NKG2DL on the NKG2DL low expressing tumor cells *in vitro*. The EMT-6 cells were stimulated with CpG ODN at 1, 3 and 6 μg/ml for 24 hours, and analyzed for their expression of RAE-1, H60 and MULT-1 by Flow cytometry. The results indicated that the NKG2DL could not be directly regulated by the CpG ODN at different doses (Figure 4A). Next, the murine lymph node cells were cultured with the CpG ODN at 6μg/ml for 48 hours for collecting supernatant. The supernatant was used to culture the EMT-6 cells for 24 hours to analyze its effect on NKG2DL expression by flow cytometry. The results showed that the CpG ODN-conditioned supernatant increased the expression of MULT-1 (*P*=0.0394), unaffected RAE-1 expression, and down-regulated H60 expression on the EMT-6 cells (*P*=0.0007) (Figure 4B). Furthermore, we detected the total H60 expressed in the EMT-6 cells by western blot and in the cells permeablized with saponin by flow cytometry, and found that the supernatant up-regulated the total H60 expression (Figure 4C and 4D). However, we kinetically tested the surface expression of H60 on EMT-6 cells stimulated by the CpG ODN-conditioned supernatant, and found that the H60 expression was increased after 2 hour stimulation (*P=0.024*), returned to the level as that in the control after 12 hour stimulation, and decreased after 24 hour stimulation (*P*=0.0056) (Figure 4E). To observe whether the surface MULT-1 up-regulated EMT-6 cells were susceptible to NK cytolysis, we cultured lymph node cells from the naive BALB/c mice with the EMT-6 cells in the presence of 6μg/ml CpG ODN for 24 hours, and found that the MULT-1 up-regulated EMT-6 cells were significantly killed at E:T ratios of 5:1 to 40:1 (Figure 4G, left panel). Alternatively, the B16 cells were used to further confirm the effect of the CpG ODN on NKG2DL up-regulation and NK cytolysis. The results showed that the CpG ODN-conditioned supernatant up-regulated the expression of MULT-1 (*P*=0.0481), not RAE-1 and H60 (Figure 4F), and the B16 cells were significantly lysed by the lymph node cells from naive C57BL/6 mice at the E:T ratio of 40:1 (Figure 4G, right panel).

### Rejection of the allogeneic tumors derived from the NKG2DL up-regulated tumor cells

**Figure 5:**
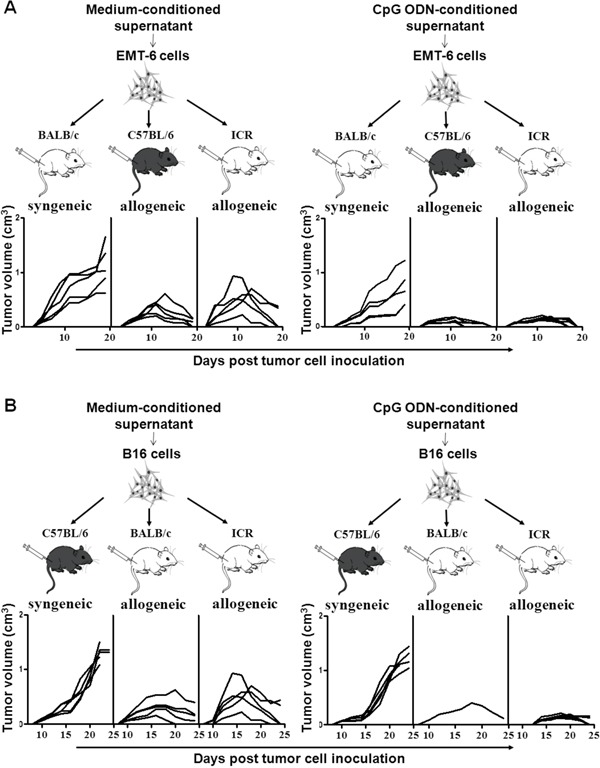
Allogeneic tumor formation of tumor cells stimulated with the CpG ODN-conditioned supernatant EMT-6 or B16 cells were cultured with or without CpG ODN-conditioned supernatant and then inoculated s.c. into the syngeneic or allogeneic mice, respectively. Tumor volume was measured every other day. Each line represents the kinetic tumor growth in each mouse. The growth curves of the tumors formed by EMT-6 cells **A.** or B16 cells **B.**

Based on the above results, we artificially up-regulated the expression of NKG2DL on the EMT-6 cells and B16 cells by culturing them with the CpG ODN-conditioned supernatant, and then inoculated the cells into the syngeneic and allogeneic mice, respectively to observe tumor development. As shown in Figure 5A, the EMT-6 cells with or without NKG2DL up-regulation were developed into palpable tumors in the allogeneic C57BL/6 and ICR mice, and in the syngeneic BALB/c mice on day 5 after the inoculation. While in allogeneic C57BL/6 mice, the NKG2DL up-regulated EMT-6 cells only formed tumors with an average volume of 150mm^3^, 1/2 smaller than that in the control mice, on day 11. On day 19, the tumors were impalpable in all the 5 mice, while still palpable in 3 out of the 5 mice in the control group. In allogeneic ICR mice, the NKG2DL up-regulated EMT-6 cells were developed into tumors with a peak average volume of 200mm^3^ on day 12 and the tumors were completely regressed in all the 5 mice on day 19. Whereas, the peak average volume of tumors was 500mm^3^ in control mice on day 9, afterwards, the tumors were regressed and impalpable in 3 out of the 5 mice on day 19. In syngeneic BALB/c mice, NKG2DL up-regulated EMT-6 cells were developed into tumors with a size exceeding 1000mm^3^ in 1 out of the 5 mice on day 19, while the tumors with the similar size occurred in 3 out of the 5 mice in control mice on the day. Similarly, as shown in Figure 5B, the B16 cells with or without NKG2DL up-regulation were all developed into palpable tumors in the allogeneic and syngeneic mice on day 10 after the inoculation. In allogeneic BALB/c mice, NKG2DL-upregulated cells were developed into tumors only in 1 out of 5 mice, and the tumors reached to a peak average volume of 405mm^3^ on day 18 and were impalpable on day 26. While the tumors were formed in all the 5 mice with the peak average volume of 330mm^3^ in the control group on day 16 and completely regressed on day 30. In allogeneic ICR mice, the NKG2DL up-regulated cells formed tumors with a peak average volume of 160mm^3^ on day 20 and the tumors were impalpable on day 26, whereas, the tumors, with a peak average volume of 500mm^3^ in control mice on day 14, were completely regressed on day 33. In syngeneic C57BL/6 mice, the growing tumors killed 3 out of the 5 control mice, while only 1 out of the 5 mice inoculated with the NKG2DL up-regulated B16 cells, on day 24.

**Figure 6:**
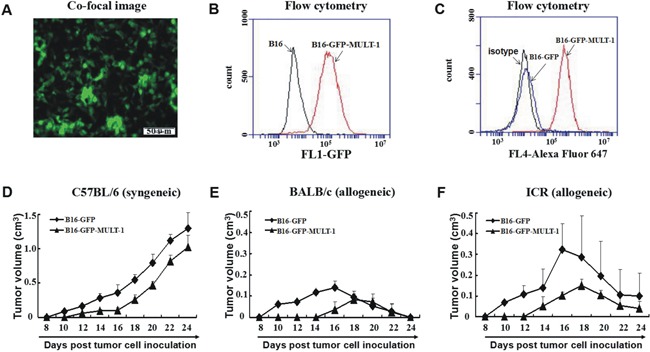
Syngeneic and allogeneic tumor formation of MULT-1 gene transfected B16 cells in mice MULT-1 gene transfected B16 cells (B16-GFP-MULT-1) were screened by bright green fluorescence under fluorescence microscope **A.** and by flow cytometry **B.**, and then confirmed using anti-MULT-1 mAb by flow cytomerty with mock-transfected B16 cells (B16-GFP) as a control **C.** The B16-GFP-MULT-1 and B16-GFP were inoculated s.c. into C57BL/6 **D.**, BALB/c **E.** and ICR mice **F.**, respectively, and the tumors were measured every other day. The tumor sizes of 5 mice per group were shown as average tumor volume (means±SEM).

To further consolidate the contribution of NKG2DL up-regulation to the rejection of allogeneic tumors, we established MULT-1 gene transfected B16 cells (B16-GFP-MULT-1) (Figure 6A-6C) and mock-transfected B16 cells (B16-GFP) (Figure 6C), and then subcutaneously inoculated the cells into the allogeneic mice and syngeneic mice, respectively, to observe tumor development. The results showed that in allogeneic BALB/c mice, the B16-GFP-MULT-1 formed palpable tumors in 3 out of the 5 mice on day 16 after the inoculation, and the tumors reached to a peak average volume of 80mm^3^ on day 18. Comparatively, the B16-GFP initiated palpable tumors in all the 5 mice on day 10, and the tumors reached to a peak average volume of 140mm^3^ on day 16. In allogeneic ICR mice, the B16-GFP-MULT-1 formed palpable tumors in all the 5 mice on day 14, and the tumors reached to a peak average volume of 150mm^3^ on day 18. While, the B16-GFP were developed into palpable tumors in all the 5 mice on day 10, and the tumors reached to a peak average size of 320mm^3^ on day 16. In syngeneic C57BL/6 mice, the tumors developed from the B16-GFP began to be palpated in all the 5 mice on day 10, continued to grow and circumvented 3 out of the 5 mice on day 24. Whereas, the B16-GFP-MULT-1 were developed into palpable tumors in all 5 mice on day 12, and all the 5 mice were still alive on day 24. To sum up, the NKG2DL up-regulation did contribute to the early rejection of the allogeneic tumors.

## DISCUSSION

Up to now, few researches have been reported about the correlation of the NKG2DL expression on tumor cells and allogeneic tumor formation at early stage after the tumor cells transplanted in mice. Our results demonstrated that NKG2DL high expressing tumor cells, such as GL261 cells (murine glioma cell line cells derived from C57BL/6 mice), could not form palpable tumors in allogeneic BALB/c mice and ICR mice, whereas the NKG2DL low expressing tumor cells, like EMT-6 cells (murine breast cancer cell line cells derived from BALB/c mice) and B16 cells (murine melanoma cell line cells derived from C57BL/6 mice), could form allogeneic tumors in the mice at the early stage, respectively (Figure 1C-1E). Comparatively, the EMT-6 cells and the B16 cells with up-regulated expression of NKG2DL induced by CpG ODN, and the B16 cells transfected with MULT-1 genes could only form considerably smaller allogeneic tumors in the mice at the early stage (Figures 5 and 6).

As to the underlined mechanisms on the early formation or rejection of the allogeneic tumors in mice, we found that the expressed NKG2DL on tumor cells, especially RAE-1, was one of the determining factors. The overwhelming expressed RAE-1, as shown in Figure 1C-1E, rendered the GL261 cells unable to form palpable subcutaneous allogeneic tumors, while barely expressed NKG2DL including RAE-1, H60 and MULT-1 allowed the EMT-6 cells and B16 cells to develop into early allogeneic tumors. Since the subcutaneous model may not be fully informative on the glioma derived GL261 cells, we used an intracranial model to further explore the correlation of RAE-1 high expression and GL261 cell rejection in brains of the allogeneic mice, and obtained the results showing that the GL261 cell inoculated allogeneic BALB/c mice and ICR mice survived significantly longer than the control syngeneic C57BL/6 mice (Supplementary Figure S2B). Consistent with the observation, RAE-1 gene-transfection was found resulted in rapid rejection of EL4 (a thymoma), RMA (a T-cell lymphoma) and B16 (a melanoma) cells in syngeneic mice; and 10-fold up-regulated RAE-1 led to less lung metastatic loci from by i.v. transfused B16 cells [11]. Intriguingly, the NKG2DL up-regulation could be achieved by some agents, such as CpG ODN. The CpG ODN administration up-regulated RAE-1, H60 and MULT-1 on breast cancer cells, H60 and MULT-1 on melanoma cells, and made them only capable of forming dramatically smaller tumors in syngeneic mice, implying that the CpG ODN induced retardation of the tumors by up-regulating NKG2DL. Noticeably, the up-regulation could be attributed to the CpG ODN induced cytokines because CpG ODN-conditioned supernatant, not CpG ODN itself, up-regulated NKG2DL expression on the EMT-6 cells and B16 cells *in vitro* (Figure 4A-4F), and the tumor cells induced enhanced rejection in allogeneic mice at the early stage (Figure 5A and 5B). The components with the NKG2DL up-regulating activity in the supernatant might be attributed to type I IFN-α/β because which was confirmed to be induced by the CpG ODN [18] and to be able to up-regulate RAE-1 [19] and MICA/B [20]. The NKG2DL induced rejection on the allogeneic tumors was further consolidated using MULT-1 gene transfected B16 cells. The reason of selecting the MULT-1 gene and the B16 cells is that the single up-regulated MULT-1 on B16 cells was found capable of inducing the rejection (Figures 4F and 5B). Similarly, we confirmed that the MULT-1 gene transfection resulted in early rejection of the allogeneic tumors (Figure 6D-6F).

As to why and how the NKG2DL expression determines the rejection or formation of the allogeneic tumors at the early stage, we found that NK cells could be the major type of NKG2D^+^ cells that mediated the rejection. NKG2D^+^ NK cells were found significantly increased in peripheral lymphoid organs of the allogeneic mice inoculated with RAE-1 high expressing GL261 cells, not NKG2DL low expressing B16 cells (Figure 2A), suggesting that the NKG2DL high expressing tumor cells could mobilize the NKG2D^+^ NK cells to eliminate the tumor cells. Because of this, at least, the GL261 cells rather than the B16 cells, failed to develop into palpable allogeneic tumors in the BALB/c mice, although both of them are C57BL/6 mouse origin. The similar phenomena were reported occurred in NKG2DL^+^ benign allogeneic grafted mouse neural precursor cells [15] and rat liver cells [21]. The allograft survival could be prolonged by depleting NK cells, indicating that NKG2D^+^ NK cells could eliminate the NKG2DL^+^ graft cells [22]. In addition to the *in vivo* data on the NKG2DL^+^ benign cells, NKG2DL high expressing glioma cells [16] and breast cancer stem cells [17] were found to be killed by allogeneic NKG2D^+^ NK cell *in vitro*. Except the activating signals primed by the NKG2DL, the inhibitory signals triggered by MHC-I molecules on the target cells also affected the NK cell activation. High MHC-I molecule expression was associated with resistance of target cells such as glioma cells to NK cell cytolysis [23]. However, in this study, we found that although expressing MHC-I molecules (Supplementary Figure S1A and S1C), the GL261 cells, murine glioma cell line cells, were still rejected at the early stage after being subcutaneously inoculated into the allogeneic mice (Figure 1C). The rejection could be associated to the highly expressed RAE-1 on the GL261 cells (Supplementary Figure S1B and S1C). The RAE-1, after engagement with NKG2D, may induce potent activating signals that over-pass the inhibitory signals induced by MHC-I molecules (Supplementary Figure S1D). Overall, in this study, we found for the first time that the level of NKG2DL determined the rejection of allogeneic tumor cells in mice at the early stage after the inoculation, and NKG2D^+^ NK cells were the potential effector cells. Noticeably, NKG2D^+^ NK cells were observed to be significantly increased in lymph nodes of the allogeneic C57 mice experienced complete regression of the tumors from EMT-6 cells (Figure 2B, upper panel), and the lymph node cells of the mice could efficiently kill RAE-1 expressing YAC-1 cells, typical target cells of murine NK cells (Figure 2D, upper panel). The results suggested that the NKG2D^+^ NK cells could be persistently existed to guarantee the immunosurveillance to the allogeneic tumor cells *in vivo*. Furthermore, NK cells could also shape the subsequent T cell immune response to allogeneic tumor cells. NK cell generated allogeneic tumor cell debris could be engulfed and presented to T cells by DC, resulting in the proliferation and killing of CTL (Figure 2D, lower panel). In addition to the NKG2D^+^ NK cells, NKG2D^+^ CD8^+^ T cells were also dramatically increased in the peripheral lymphoid organs of the allogeneic BALB/C mice inoculated with NKG2DL high expressing GL261 cells (Figure 2A). The increased NKG2D^+^ CD8^+^ T cells may also participate in the rejection of allogeneic GL261 cells at the early stage. Consistently, *ex vivo* expanded NKG2D^+^ CD8^+^ T cells isolated from myeloma patients were potent at recognizing and killing NKG2DL high expressing allogeneic myeloma cells [24]. Besides, the expanded CD8^+^ T cells expressed up-regulated NKG2D [25] and could reinforce the clearance of RAE-1 expressing leukemia cells in mice [26].

With the technical development of *ex vivo* expansion of NK cells from healthy donors [27], adaptive transfer of allogeneic NK cells has been increasingly tested for treating patients with non-small cell lung cancer [28, 29], acute myeloid leukemia [30], ovarian cancer [31, 32] and malignant lymphoma [33]. Promisingly, the present study could provide insights for combining the allogeneic NK cells with various NKG2DL inducers to reinforce the efficacy of the allogeneic NK cell-based anti tumor therapy, and the CpG ODN could offer an option as this kind of inducer. Noticeably, spironolactone, an FDA-approved diuretic drug, was demonstrated to enhance allogeneic NK cell efficacy in treating osteosarcoma in mice by up-regulating NKG2DL expression [34, 35].

## MATERIALS AND METHODS

### Cells and cell lines

Lymph node cells were isolated from bilateral axillary, inguinal and popliteal lymph nodes of euthanized mice and splenocytes were obtained from spleens of the mice by lysing erythrocytes with lysis buffer (10mM KHCO_3_, 150mM NH_4_Cl, 10mM EDTA, PH7.4). BALB/c mice-derived EMT-6 breast cancer cells (EMT-6), C57BL/6 mice-derived B16 melanoma cells (B16) and C57BL/6 mice-derived GL261 glioma cells (GL261) (American Type Culture Collection) were maintained in RPMI 1640 supplemented with 10% (V/V) fetal bovine serum (FBS) (GIBCO) and antibiotics (100IU of penicillin/ml and 100IU of streptomycin/ml). All cells were cultured at 37°C in a 5% CO_2_ humidified incubator.

### Mice

Female BALB/c, C57BL/6 and ICR mice, 6 to 8-week-old, were purchased from the Experimental Animal Center, Medical College of Norman Bethune, Jilin University (Changchun, China), and maintained in laminar flow rooms and used for experiments in accordance with the National Institute of Health Guide for the Care and Use of Laboratory Animals, and with the approval of the Scientific Investigation Board of Science & Technology of Jilin Province.

### Antibodies and reagents

The following antibodies were from BD biosciences: FITC Rat anti-mouse CD4 (553651), FITC Rat anti-mouse CD8a (553031), FITC-conjugated hamster anti-mouse CD11c (553801), FITC Rat anti-mouse CD19 (553785), PE anti-mouse CD25 (553075), FITC Rat anti-mouse CD49b (561067), PE Hamster anti-mouse CD69 (553237), APC Rat anti-mouse CD86 (558703), PE Rat anti-mouse CD314 (558403), FITC labeled anti-mouse NK1.1 (553164), Alexa Fluor 647 Rat anti-mouse Foxp3 (560402) and FITC Rat anti-mouse H-2K^b^ (553569). The following antibodies were from R & D system: Rat anti mouse RAE-1 (MAB17581), Rat anti mouse H60 (MAB1155), Rat anti mouse MULT-1 (MAB2588). FITC goat anti-Rat (A24544) and Alexa Fluor 647 Goat anti-Rat (bs-0293G-AF647) were obtained from Novex and BIOSYNTHESIS, respectively. A CpG ODN (5'-TCGCGAACGTTCGCCGCGTTCGAACGCGG-3′) with full-phosphorothioate modification was synthesized in Takara Biotechnology Company (Dalian, China) and diluted in PBS buffer with no detectable endototin (Limulus amebocyte lysate assay, Associates of Cape Cod, Inc.). CpG ODN-conditioned supernatant was prepared by culturing mouse lymph node cells with CpG ODN at 6μg/ml for 48 hours and collecting the culture medium. All reagents used in this study were pyrogen-free.

### Animal experiments

For development of subcutaneous allogeneic tumors, BALB/c mice, C57BL/6 mice and ICR mice were inoculated with 1×10^6^ EMT-6 cells, B16 cells, GL261 cells, B16-GFP-MULT-1 and B16-GFP in 0.2 ml serum-free medium s.c. at the right back near hind leg on day 0, respectively. The developed tumors were measured every other day and the tumor size was expressed as tumor volume (cm^3^). Tumor volume=length×width^2^×0.5. For CpG ODN treatment, BALB/c mice or C57BL/6 mice were s.c. injected with 5×10^5^ EMT-6 cells or 2×10^5^ B16 cells in 0.2 ml serum-free medium at the right back near hind leg on day 0 and from day 8 when the tumors could be palpated, the mice were treated by CpG ODN at 25μg per mouse through s.c. injection at right inguinal lymph node area for six times in one-day interval. Tumor volume was measured every other day.

For the establishment of intracranial glioma, BALB/c mice, C57BL/6 mice and ICR mice were intracranially (IC) injected with GL261 (2×10^4^/mouse) cells on day 0, respectively. In brief, the GL261 cells were suspended in 5μl of PBS and stereotactically injected using a 10 ml Hamilton syringe through an entry site at the bregma 2mm to the right of the sagittal suture, 3mm below the surface of the skull of anesthetized mice using a Kopf stereotactic frame (Kopf Instruments). After the injection, any mouse showing any pathologic signs caused by the surgery, such as hemi-paresis, loss of appetite, or any altered grooming habits, was excluded from the experiment. The body weights were measured every other day until the first mouse was dead, and the mice were monitored daily for manifesting any pathologic signs. The animal experimental manipulation was performed according to the National Institute of Health Guide for the Care and Use of Laboratory Animals, with the approval of the Scientific Investigation Board of Science and Technology of Jilin Province.

### Histologic analysis

The brains of the dead mice intracranially inoculated with the GL261 cells were fixed in 4% (wt/vol) paraformaldehyde, embedded into paraffin, sectioned, stained with hematoxylin and eosin (H & E), and observed under the microscope.

### Flow cytometry

For direct staining, the lymph node cells and splenocytes were stained with fluorescence-conjugated mAbs against CD4, CD8, CD11c, CD19, CD25, CD49b, CD86, CD314, NK1.1, Foxp3, and GL261 cells were stained with fluorescence-conjugated mAbs against H-2K^b^ molecules, respectively, for 30 minutes on ice in dark, followed by washing twice with cold PBS. For indirect staining, EMT-6 cells, B16 cells and GL261 cells were collected and stained with anti-RAE-1, -H60 and -MULT-1 mAb, respectively, for one hour on ice in dark, followed by washing twice with cold PBS, and then incubated with FITC-conjugated secondary antibodies for 30 minutes on ice in dark followed by washing twice with cold PBS. And the B16-GFP-MULT-1 and B16-GFP were stained with anti-MULT-1 mAb followed by Alexa Fluor 647-conjugated secondary antibodies. For H60 intracellular staining, the EMT-6 cells were treated with/without CpG ODN-conditioned supernatant, fixed with 4% paraformaldehyde and permeablized with 0.1% saponin, followed by staining using anti-H60 mAb as described above. All stained cells were analyzed by Accuri C6 (BD) and Cyto FLEX (BECKMAN COULTER). Live cells were carefully gated by forward and side scattering.

### RNA isolation and real-time RT-PCR

Total RNA was isolated from GL261 cells with trizol reagent and reverse transcribed using cDNA Synthesis Kit. Quantitative real-time PCR (qRT-PCR) was performed using Two-step SYBR green qPCR assays and the target mRNA were identified by the specific primers as follows: MHC-I, forward:TACCTGAAGAACGGG AACGC; reverse: CCATTCAACTGCCAGGTCAG; RAE-1, forward: AAGGCAGCAGTGACCAAG; reverse: GAGAGTGTGCATCATCCAG; GAPDH, forward: ATCACCATCTTCCAGGAGCGA; reverse: TCTCGTGGTTCACACC CATCA. The data were acquired using the Step One™ real time PCR system (Applied Biosystems). The procedure of the target mRNA amplification was as follows: 1 cycle at 95°C (30 seconds) followed by 40 cycles at 95°C (5 seconds) and 64°C (31 seconds). Each assay plate included negative controls with no template. The relative amount of gene expression was analyzed with 2^−ΔΔCt^ method.

### Assessment of the NKG2DL expression on tumor cells *ex vivo*

BALB/c mice or C57BL/6 mice were injected with EMT-6 cells or B16 cells and treated by CpG ODN for six times as presented above. After 48 hours of the last injection, the BALB/c mice or C57BL/6 mice were sacrificed for isolating the tumors. The tumors were physically dissociated to achieve single-cell suspension, and then 5×10^5^ of the tumor cells in suspension were labeled with anti-RAE-1, -H60 and -MULT-1 mAb by using the indirect staining and analyzed by flow cytometry.

### *In vitro* cytotoxicity assays

1×10^5^ EMT-6 cells or B16 cells, used as target cells (T), were cultured in 96-well plates for 12 hours to confluence, and then co-cultured with lymph node cells, used as effector cells (E), isolated from naive BALB/c or C57BL/6 mice with/without CpG ODN (6μg/ml) for 24 hours at the E/T ratio of 40:1, 20:1, 10:1, 5:1. In parallel, 1×10^5^ EMT-6 cells or YAC-1 cells were incubated with the lymph node cells from BALB/c mice with complete regression of EMT-6 cell derived allogeneic tumors for 24 hours at the same E/T ratio mentioned above. The co-cultured cells were stained with propidium iodide (PI) for 20 min at room temperature and then percent cell death was calculated as (specific OD value - spontaneous OD value) / (total OD value − spontaneous OD value) × 100%.

### Western blotting

The EMT-6 cells were incubated with or without CpG ODN-conditioned supernatant for 24 hours, washed with PBS, harvested and lysed in ice-cold RIPA buffer (150 mM NaCl, 50 mM Tris, pH 7.4, 1% NP-40, 0.5% sodium deoxycholate and 0.1% SDS). The cell lysate were centrifuged (14,500g for 20 min at 4°C) and the supernatant was collected, quantified using a BCA protein assay kit (Wanleibio, Shenyang, China) and separated by 12% SDS-PAGE. Then the separated protein bands were transferred to polyvinylidene difluoride membranes (Millipore, USA). The membranes were blocked overnight at 4°C with Tris-buffered saline containing 5% nonfat dried milk and then incubated with anti-H60 mAb (R&D system, USA) or anti-GAPDH mAb (Proteintech, USA). Blots were then incubated with horseradish peroxidase (HRP)-conjugated goat anti-mouse IgG for 1 h at room temperature (Jackson immunoresearch laboratories, USA). Immunoreactive bands were visualized with SuperSignal West Pico chemiluminescent substrate (Thermo, USA).

### Establishment of MULT-1 gene trasfected B16 tumor cell line

MULT-1 encoding gene was amplified with GL261 cell derived cDNAs as templates using specific primers (upper primers: GGATATCATGGAGCTGACTGCCAGTAAC; lower primers: CTCGAGTCATGGGATCCCATCAATATCG), and cloned into downstream of green fluorescence protein (GFP) coding sequence in pcDNA3 plasmid (Invitrogen). After being confirmed by sequencing, the resultant plasmid (pcDNA3-GFP-MULT-1) and GFP gene carrying plasmid (PcDNA3-GFP) were stably transfected into murine B16 melanoma cells, respectively. The transfected cells were selected by limited dilution in RPMI1640 medium (Gibco) containing neomycin (G418) and 10% FCS (Invitrogen), identified by green fluorescence under fluorescence microscope and by flow cytometry, and confirmed by flow cytomerty using anti-MULT-1 mAb followed by Alexa Fluor 647-conjugated secondary antibodies. The MULT-1 gene transfected B16 cells (B16-GFP-MULT-1) and mock-transfected B16 cells (B16-GFP) were stored in liquid nitrogen and used to inoculate mice.

### Statistical analysis

Comparisons between groups were conducted using analysis of unpaired t tests. P value of less than 0.05 (95% CI) was considered to be statistically significant. Statistics were analyzed using Graphpad Prism 5.0 for Windows (San Diego, CA).

## SUPPLEMENTARY FIGURES


