# Supplementary material for: Attribution of NKG2DL to the inhibition of early stage allogeneic tumors in mice
Source: Oncotarget. 2016 Jul 19;7(50):82369–83. doi: 10.18632/oncotarget.10693 (PMC5347697; doi:10.18632/oncotarget.10693)
Supplement: Supplementary file 1 [file oncotarget-07-82369-s001.pdf]

Attribution of NKG2DL to the inhibition of early stage allogeneic tumors in mice

SUPPLEMENTARY FIGURES

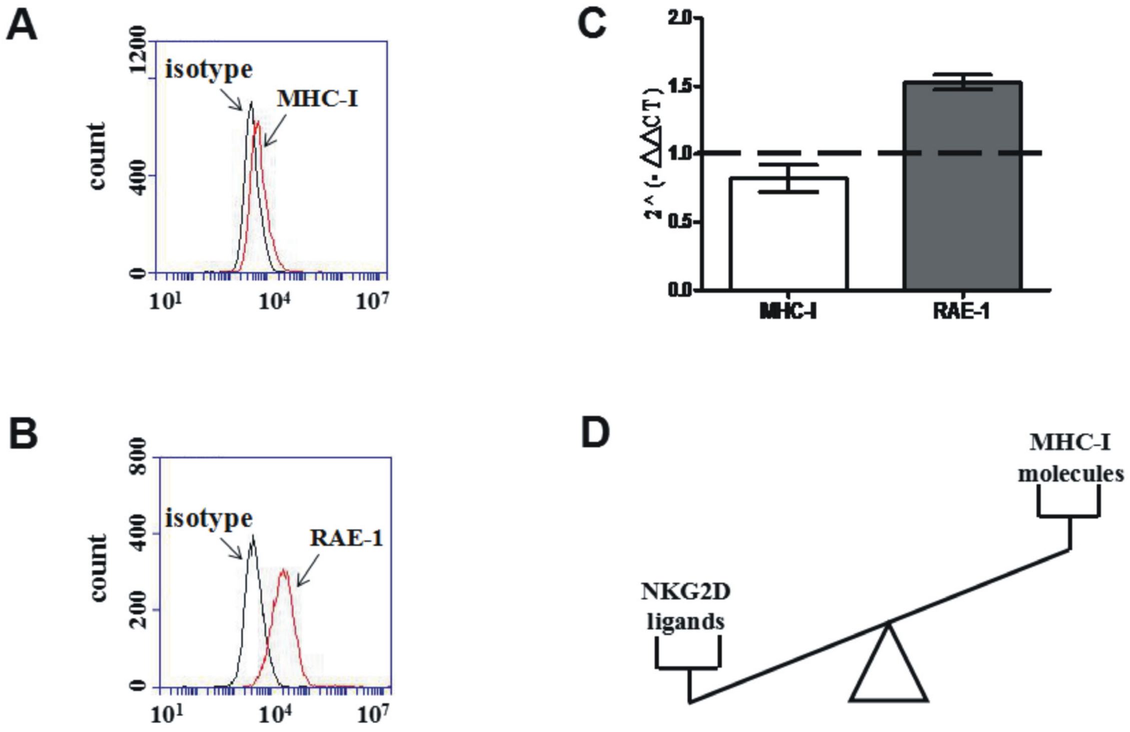

Supplementary Figure S1: The expression of RAE-1 and MHC-I molecules of GL261 cells at protein and mRNA levels. GL261 cells were stained with FITC-labeled mAb of anti-H-2K^{b} to detect MHC class I protein expression **A.** or with anti-RAE-1Ab plus FITC-labeled secondary antibodies to detect RAE-1 protein expression **B.** by flow cytometry. In parallel, the total mRNA were isolated from the cells and reverse transcribed into total cDNA from which the MHC-I and RAE-1 specific cDNA were amplified by qPCR using H2-K^{1} and RAE-1 specific primers. The graphical presentation **C.** of the pooled values for mRNA expression was from three independent experiments, and each bar represents as means±SEM. **D.** NK cell mediated killing of GL261 cells could be determined by the balance of NKG2DL induced activating signals and MHC-I molecule induced inhibitory signals.

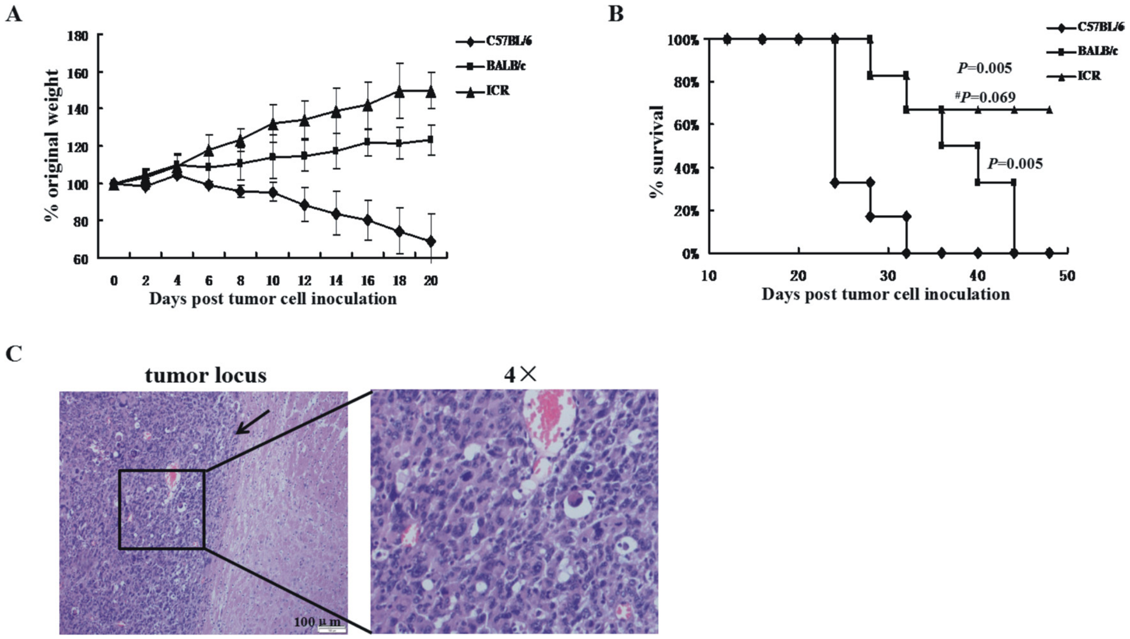

Supplementary Figure S2: The body weights, survivals and the brain tissue pathological changes of syngeneic and allogeneic mice with intracranially inoculated GL261 cells. C57BL/6, BALB/c and ICR mice were intracranially inoculated with 2×10^4 GL261 cells on day 0, respectively, and their body weights were measured every other day until the first mouse was dead and survivals were monitored daily until day 50. The dead mice were autopsied for examining the glioma cells in brains. **A.** Body weight. **B.** Survivals. *P*: versus C57BL/6 mice, $^{\#}P$: versus BALB/c mice. **C.** Micrographs of H & E staining of the brain section (400×). A representative tumor locus was shown and bounded by the normal tissue as indicated with an arrow (left). A region of the tumor locus was highlighted, magnified and shown (right).
